# Targeting Stress and the Gut–Brain Axis: Effects of Lifestyle and Psychobiotic Interventions on the Gut Microbiome in Adults—A Systematic Review of Randomized Controlled Trials

**DOI:** 10.3390/nu18172909

**Published:** 2026-09-04

**Authors:** Stavroula Asimakopoulou, Panagiotis Pipelias, Evanthia Kassi, Evangelos Oikonomou, Gerasimos Siasos, Christina Darviri

**Affiliations:** 1 Postgraduate Program “Cardiometabolic Medicine”, School of Medicine, National and Kapodistrian University of Athens, 115 27 Athens, Greece; stavasim@med.uoa.gr (S.A.); gsiasos@med.uoa.gr (G.S.); 2 Postgraduate Program “The Science of Stress and Health Promotion”, School of Medicine, National and Kapodistrian University of Athens, 115 27 Athens, Greece; cdarviri@med.uoa.gr; 3 Division of Diabetes, Endocrinology and Metabolic Diseases, First Department of Propaedeutic and Internal Medicine, Center of Expertise for Rare Endocrine Diseases (ENDO-ERN Accredited), School of Medicine, Laikon General Hospital, National and Kapodistrian University of Athens, 115 27 Athens, Greece; ekassi@med.uoa.gr; 4 Department of Biological Chemistry, School of Medicine, National and Kapodistrian University of Athens, 115 27 Athens, Greece; 5 Third Department of Cardiology, Sotiria Chest Disease General Hospital, School of Medicine, National and Kapodistrian University of Athens, 115 27 Athens, Greece; boikono@med.uoa.gr

**Keywords:** stress, gut microbiome, gut–brain axis, psychobiotics, lifestyle interventions, diet, probiotics, mindfulness, adults, randomized controlled trials

## Abstract

**Background/Objectives:** Stress is increasingly recognized as an important modulator of gut microbiome composition and function through the bidirectional microbiota–gut–brain axis. Dietary, psychobiotic, and other lifestyle-oriented interventions have therefore attracted growing interest as potential strategies for simultaneously influencing stress-related outcomes and the gut microbiome. This systematic review aimed to evaluate randomized controlled trials (RCTs) investigating the effects of lifestyle-oriented and psychobiotic interventions on both stress-related outcomes and the gut microbiome in adults. **Methods:** A systematic literature search was conducted in PubMed, Scopus, and Cochrane CENTRAL for English-language RCTs published between 2016 and April 2026, in accordance with the Preferred Reporting Items for Systematic Reviews and Meta-Analyses (PRISMA) 2020 guidelines. Eligible studies included adults (≥18 years), evaluated lifestyle-oriented or psychobiotic interventions, and reported both gut microbiome-related and stress-related psychological or biological outcomes. Risk of bias was assessed using the Cochrane Risk of Bias 2 (RoB 2) tool. Owing to substantial methodological and clinical heterogeneity, findings were synthesized narratively. **Results:** Twenty-nine RCTs met the eligibility criteria. Interventions included psychobiotic supplementation, dietary interventions and functional foods, and other lifestyle-oriented approaches. Across intervention categories, microbiome responses were heterogeneous, and no single taxonomic or diversity signature consistently characterized trials reporting favorable stress-related outcomes. Nevertheless, recurrent changes involving *Bifidobacterium*/Bifidobacteriaceae, *Lactobacillus*/Lactobacillaceae, *Faecalibacterium*, *Roseburia*, and members of the Lachnospiraceae/Ruminococcaceae families were identified across several interventions, together with changes in SCFA-related microbial features. Changes in global microbial diversity were inconsistent, and psychological and microbiome responses did not invariably occur in parallel. Overall, the randomized evidence indicates recurrent taxonomic and functional signals rather than a uniform microbiome response across stress-targeting interventions. Substantial heterogeneity in populations, intervention characteristics, study duration, outcome measures, and microbiome assessment methods limited direct comparisons across trials. **Conclusions:** Current randomized evidence does not identify a uniform microbiome signature associated with successful stress-targeting interventions but suggests partial convergence involving selected bacterial taxa and functional microbial features. The lack of consistent parallel changes in psychological and microbiome outcomes precludes conclusions regarding whether microbiome alterations mediate improvements in stress-related outcomes. Larger, longer-term RCTs incorporating standardized microbiome methodologies and functional microbial analyses are needed to determine the reproducibility and mechanistic relevance of these signals.

## 1. Introduction

Psychological stress arises when perceived environmental demands exceed an individual’s adaptive resources, eliciting psychological and biological responses that may disrupt physiological homeostasis [1]. While acute stress supports adaptation, persistent or excessive stress may contribute to neuroendocrine, immune, metabolic, and behavioral dysregulation [2]. Within the gut–brain axis, psychological stress may also influence gastrointestinal physiology and gut microbial homeostasis through interconnected neuroendocrine, neural, immune, and inflammatory pathways [3].

The hypothalamic–pituitary–adrenal (HPA) axis provides a key neuroendocrine interface between psychological stress and the gut microbial environment. Sustained HPA-axis activation and glucocorticoid exposure may alter intestinal permeability, immune signaling, and gastrointestinal physiology, thereby providing a biologically plausible pathway through which stress may influence gut microbiome composition and function [4,5,6]. However, whether modulation of this stress-related pathway is accompanied by reproducible microbiome changes in human intervention studies remains uncertain.

Human studies further suggest that stress-related neuroendocrine activity may be accompanied by changes in the gut microbial environment. In university students exposed to examination stress, increased cortisol concentrations coincided with gastrointestinal and microbial alterations that were partially attenuated following probiotic administration [7], while targeted probiotic supplementation has been associated with reduced cortisol concentrations and improved sleep quality in stressed adults [8]. These findings suggest that stress-related physiological and microbial responses may be modifiable, although gut–brain communication also involves neural pathways, particularly autonomic and vagal signaling [9,10].

The gut–brain axis is a bidirectional network integrating neural, endocrine, immune, and metabolic signaling [11]. Communication occurs through interconnected pathways including the autonomic and enteric nervous systems, with the vagus nerve serving as a major neural route [12], while HPA-axis activity provides an important neuroendocrine link between psychological stress and gastrointestinal function [6]. Together, these pathways enable reciprocal communication between psychological states, gastrointestinal physiology, and the gut microbial environment.

The gastrointestinal tract is a metabolically and endocrinologically active system that produces or regulates neuroactive compounds, including serotonin and γ-aminobutyric acid (GABA), while gut microorganisms participate in the metabolism of neurotransmitter precursors such as tryptophan [6,13,14,15]. Preliminary intervention studies suggest that specific probiotics and fermented-food interventions may influence neuroactive pathways relevant to psychological functioning; for example, *Weizmannia coagulans* supplementation has been associated with GABA-related signaling and reduced anxiety symptoms [16], while fermented-food interventions have been linked to neurochemical changes relevant to emotional regulation [17]. Although the clinical significance and causal pathways underlying these observations remain uncertain, they provide biological plausibility for interactions between microbial activity and psychological functioning.

Immune signaling is an important component of gut–brain communication, as changes in gut microbial composition may influence intestinal barrier integrity and inflammatory activity, while immune mediators can affect both gastrointestinal and brain function [18,19]. The gut microbiome contributes to mucosal integrity, metabolic activity, and immune homeostasis [10,20], whereas dysbiosis has been associated with increased intestinal permeability, inflammatory activation, and altered neuroactive signaling [21,22]. These interactions provide an additional biological pathway through which stress and microbiome-related processes may be interconnected.

Short-chain fatty acids (SCFAs), particularly acetate, propionate, and butyrate, are key microbial metabolites produced through bacterial fermentation of non-digestible carbohydrates and dietary fiber [23]. SCFAs contribute to epithelial integrity and reduced intestinal permeability [21,24] and exert immunomodulatory effects through the regulation of inflammatory responses [25,26]. Conversely, reduced SCFA production in the context of dysbiosis has been associated with low-grade systemic inflammation relevant to metabolic and psychiatric disorders [27,28]. These findings highlight microbial metabolic function, in addition to taxonomic composition, as a potentially relevant dimension of the stress–microbiome relationship.

The vagus nerve provides an additional pathway for bidirectional gut–brain communication through neural and immunoregulatory signaling [29,30]. Vagal signaling may facilitate interactions among microbial metabolites, immune processes, and the brain, while impaired vagal function has been associated with increased stress and inflammatory activity [31,32]. These observations support the biological plausibility of vagal involvement in stress–microbiome interactions, although its contribution to intervention-related microbiome and psychological changes remains uncertain.

The clinical consequences of prolonged stress extend beyond psychological symptoms, as sustained HPA-axis activation and chronic stress have been associated with immune, neurological, metabolic, and cardiometabolic disturbances [33,34,35]. Stress may also exacerbate gastrointestinal disorders such as irritable bowel syndrome, where altered intestinal permeability and low-grade inflammation are commonly observed [36,37]. These interactions may contribute to a reciprocal relationship in which stress-related physiological changes influence the gut microbial environment, while microbial and immune signaling may, in turn, affect psychological and gastrointestinal functioning [6]. This bidirectional relationship provides a rationale for investigating non-pharmacological interventions that may influence both stress-related outcomes and the gut microbiome.

Diet is an important modifiable determinant of gut microbiome composition and function and may also influence physiological responses to psychological stress [38,39]. Plant-rich, high-fiber dietary patterns, including the Mediterranean diet, have been associated with greater microbial diversity and enrichment of SCFA-producing taxa [40], whose metabolites may support intestinal barrier integrity and inflammatory regulation [23]. Conversely, Western-style dietary patterns have been associated with dysbiosis and increased inflammatory activity [41]. Specific fiber-, polyphenol-, and bioactive-rich foods may also influence stress-related microbial responses [42], with walnut consumption investigated for its potential to preserve microbial diversity and support gut–brain axis function during stress [43].

Increasing attention has been directed toward dietary approaches designed to modulate the gut microbiome, including psychobiotic diets rich in prebiotic fibers and fermented foods. A psychobiotic dietary intervention has been associated with reduced perceived stress and changes in microbiota-related metabolic pathways, including tryptophan metabolism [3], while Mediterranean dietary interventions have been associated with stress-related and microbial outcomes in vulnerable populations, including pregnant women [44]. Together, these findings suggest that broader dietary patterns may influence both gut microbiome characteristics and stress-related outcomes, although the specific microbial mechanisms underlying these associations remain uncertain.

Probiotic and prebiotic supplementation provides a targeted approach to gut microbiome modulation. Although not lifestyle behaviors per se, these interventions are frequently incorporated into lifestyle-oriented and psychobiotic strategies because they directly target the gut microbial environment. Their effects on stress-related, neuroendocrine, sleep, and microbiome outcomes may vary according to strain or formulation, dose, intervention duration, baseline microbiome characteristics, health status, and dietary context; therefore, findings from individual probiotic formulations should not be generalized across products.

Physical activity may influence both neuroendocrine regulation and the gut microbiome [45]. Regular exercise has been associated with greater microbial diversity and enrichment of SCFA-producing taxa, including *Faecalibacterium prausnitzii* and members of the Lachnospiraceae family, although these associations may be influenced by diet and other lifestyle factors [46]. Exercise may also modulate stress-related HPA-axis responsiveness and cortisol dynamics [47]. However, intervention-based evidence simultaneously evaluating exercise-related changes in both stress and microbiome outcomes remains limited.

Sleep is closely linked to stress regulation and gut microbial homeostasis. Poor sleep has been associated with HPA-axis dysregulation and altered cortisol secretion [48,49], as well as reduced microbial diversity, dysbiosis, and inflammatory and metabolic disturbances [50]. Conversely, microbiome-targeted interventions, including multispecies and specific probiotic formulations, have been associated with improvements in sleep quality and reductions in perceived stress or cortisol concentrations [51,52]. However, whether improvements in sleep are attributable to microbiome alterations or to broader effects on stress regulation remains unclear.

Mindfulness-based interventions have demonstrated beneficial effects on perceived stress and related psychological outcomes [53,54], potentially through modulation of HPA-axis activity, cortisol secretion, autonomic regulation, and vagal signaling [55]. Preliminary evidence suggests that stress-reduction practices may also be accompanied by changes in gut microbial composition [56], with mindfulness-, yoga-, and mindfulness-based cognitive interventions associated with concurrent psychological and microbial changes in populations including individuals with irritable bowel syndrome or elevated anxiety [57,58]. However, the mechanisms linking psychological interventions with microbiome alterations remain uncertain and may involve interacting neural, neuroendocrine, immune, dietary, and behavioral pathways [59].

Despite the growing number of studies examining psychological stress, the gut microbiome, and lifestyle-oriented interventions, several important gaps remain. Observational studies cannot determine whether microbiome alterations are a cause, consequence, or correlate of psychological stress, while intervention studies have not established whether improvements in stress-related outcomes are accompanied by reproducible changes in gut microbial composition or function. In particular, it remains unclear whether different stress-targeting interventions produce shared taxonomic or functional microbiome responses, whether changes in microbial diversity consistently parallel psychological improvement, and whether recurrent microbial alterations extend beyond those expected from direct probiotic administration. Substantial heterogeneity in study populations, intervention types and durations, microbiome assessment methods, stress measures, and reported outcomes has further limited cross-study comparisons and the identification of reproducible microbiome-related patterns across randomized trials.

A focused synthesis of randomized controlled trials is therefore needed to determine whether interventions targeting psychological stress are accompanied by reproducible changes in gut microbiome composition, diversity, or function. Although dietary, behavioral, and psychobiotic interventions differ in their mode of delivery, they were evaluated within a common framework because each may influence stress-related physiology while directly or indirectly modifying the gut microbial environment. Accordingly, the primary aim of this systematic review was to assess the effects of stress-targeting interventions—including dietary, physical activity, sleep-related, mindfulness-based, and probiotic or prebiotic approaches—on gut microbiome composition, diversity, and functional characteristics in adults. Secondary objectives were to summarize concurrent changes in perceived stress and related psychological outcomes, examine available neuroendocrine and inflammatory biomarkers, and compare microbiome-related findings across intervention categories.

## 2. Materials and Methods

### 2.1. Study Design

This systematic review was conducted to synthesize the available evidence regarding the effects of lifestyle-oriented and psychobiotic interventions on gut microbiome composition, diversity, and function in adults in relation to psychological stress. The review was conducted and reported in accordance with the Preferred Reporting Items for Systematic Reviews and Meta-Analyses (PRISMA) statement [60]. The PRISMA 2020 checklist is provided in Supplementary Table S1. The review protocol was not prospectively registered, and no registration number is therefore available.

### 2.2. Search Strategy

A systematic literature search was conducted in PubMed, Scopus, and the Cochrane Central Register of Controlled Trials (CENTRAL). The final search was performed on 26 April 2026 and included studies published from 1 January 2016 to 26 April 2026. The 2016–2026 publication window was selected to capture contemporary randomized evidence reflecting current microbiome sequencing methodologies and modern lifestyle- and microbiome-targeted intervention approaches.

The search strategy was designed to identify RCTs investigating the effects of lifestyle-oriented and psychobiotic interventions on the gut microbiome in adults experiencing psychological stress or related psychological outcomes. Search terms covered four main concepts: (i) psychological stress, (ii) the gut microbiome, (iii) randomized intervention studies, and (iv) lifestyle interventions.

The principal search terms included “stress”, “psychological stress”, “perceived stress”, “gut microbiome”, “gut microbiota”, “intestinal microbiota”, “randomized”, “intervention”, “trial”, “diet”, “Mediterranean diet”, “exercise”, “physical activity”, “mindfulness”, “MBSR”, “sleep intervention”, “probiotics”, and “prebiotics”. Terms were combined using the Boolean operators AND and OR. The search strategy was adapted to the indexing system and search syntax of each database. The complete database-specific search strategies, including the exact search strings and limits applied, are provided in Supplementary Table S2.

Searches were restricted to studies published in English involving human adults (≥18 years) and randomized controlled trials published between 2016 and 2026.

### 2.3. Eligibility Criteria

Eligibility criteria were established according to the Population, Intervention, Comparator, Outcomes (PICO) framework (Table 1) [61].

The randomized trial, rather than the individual publication, was considered the primary unit of eligibility. When multiple publications reported complementary outcomes from the same randomized trial, these reports were considered jointly to determine eligibility and to provide a complete characterization of the intervention effects. Accordingly, a microbiome-focused secondary or subanalysis publication could be retained when the corresponding parent randomized trial reported an eligible stress-related psychological or biological outcome, provided that the publications referred to the same randomized intervention and study population.

Inclusion Criteria:

Studies were included if they met all of the following criteria: involved human adults aged ≥18 years; used a randomized controlled trial design; evaluated a lifestyle-oriented or psychobiotic intervention; included an appropriate comparison group; provided both gut microbiome outcomes and stress-related psychological or biological outcomes, either within the same publication or across clearly linked reports arising from the same randomized trial; were published in English from 1 January 2016 to 26 April 2026; and reported original research findings.

Exclusion Criteria:

Studies were excluded if they involved animal models or in vitro experiments; used observational, cross-sectional, case–control, cohort, or other non-interventional designs; were review articles, systematic reviews, meta-analyses, conference abstracts, study protocols without published results, editorials, or letters; included exclusively pediatric or adolescent populations (<18 years); did not evaluate gut microbiome outcomes; did not assess a predefined stress-related psychological or biological outcome; or had been formally retracted.

### 2.4. Study Selection

**Figure 1:**
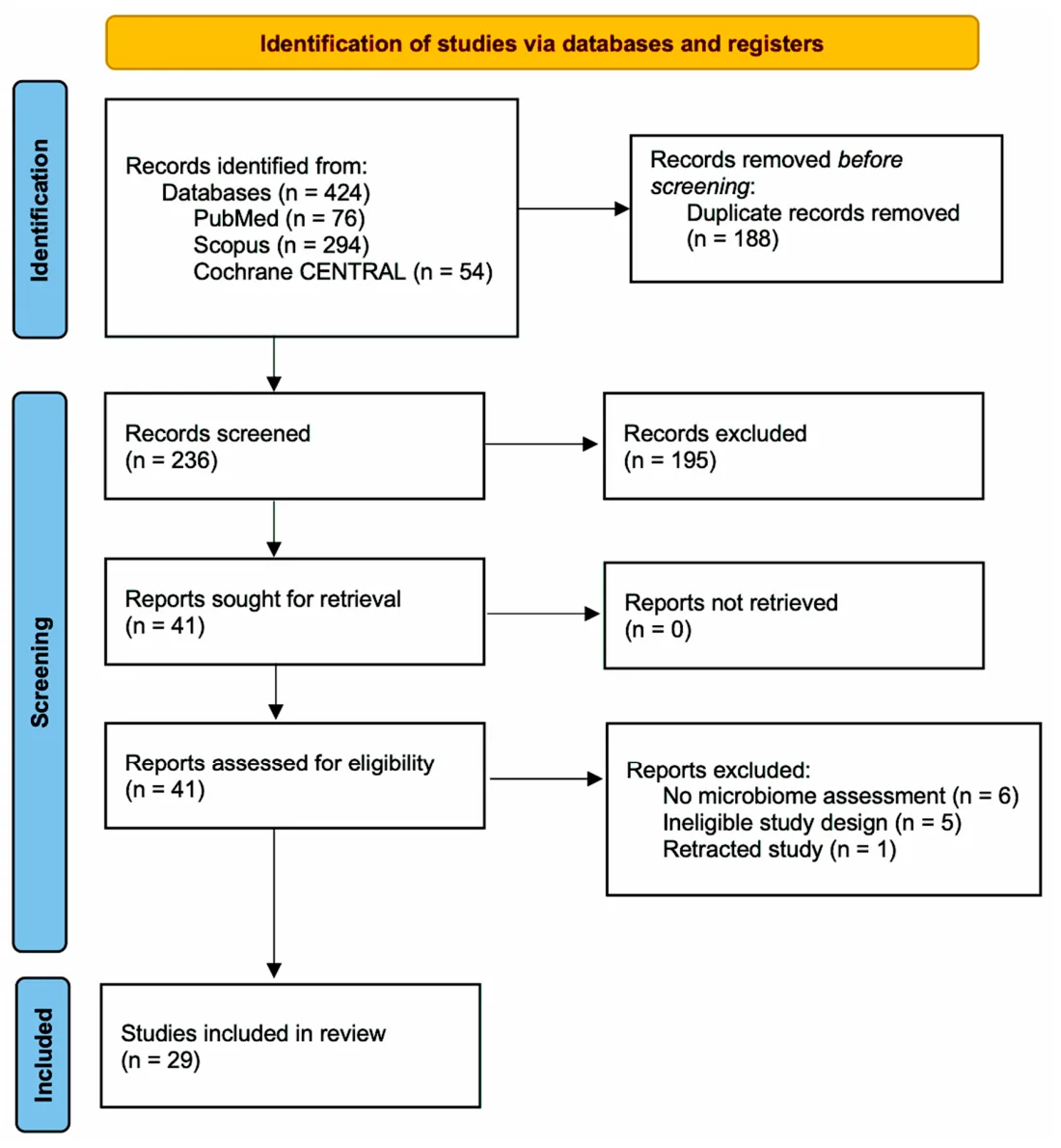
Study selection process according to PRISMA 2020 flow diagram [60].

Records identified through the database searches were imported and duplicate records were removed prior to screening. Titles and abstracts were independently screened by two reviewers (S.A. and P.P.) according to the predefined eligibility criteria. Potentially eligible articles subsequently underwent full-text assessment by the same reviewers. Disagreements arising during either stage of the selection process were resolved through discussion. When consensus could not be reached, a third reviewer was consulted to reach the final decision. The study selection process is summarized in the PRISMA flow diagram (Figure 1).

### 2.5. Data Extraction

Data were extracted independently by two reviewers (S.A. and P.P.) using a predefined data extraction form. Discrepancies were resolved through discussion until consensus was achieved. The following information was extracted from each included study: first author and year of publication; study population and participant characteristics; sample size, age, and health status; intervention type, composition, duration, and frequency; characteristics of the comparator group; gut microbiome assessment methodology; microbial diversity and composition outcomes; changes in the abundance of bacterial taxa; psychological outcomes, including perceived stress, anxiety, depressive symptoms, and sleep quality; neuroendocrine and inflammatory biomarkers; cardiometabolic outcomes. Extracted studies were subsequently categorized according to intervention type to facilitate narrative synthesis.

### 2.6. Risk-of-Bias Assessment

The methodological quality and risk of bias of the included randomized controlled trials were independently evaluated by two reviewers (S.A. and P.P.) using the revised Cochrane Risk of Bias tool for randomized trials (RoB 2) [62]. The assessment included the following domains: bias arising from the randomization process; bias due to deviations from intended interventions; bias due to missing outcome data; bias in the measurement of the outcome; and bias in the selection of the reported result. Disagreements between reviewers were resolved through discussion until consensus was achieved. Each domain was judged as low risk of bias, some concerns, or high risk of bias, and an overall risk-of-bias judgment was assigned according to the RoB 2 guidance.

### 2.7. Data Synthesis

Because substantial methodological and clinical heterogeneity was anticipated across study populations, intervention characteristics, intervention duration, microbiome assessment methods, and reported outcomes, a qualitative narrative synthesis was performed rather than a meta-analysis. Accordingly, no pooled effect measures were calculated. No statistical transformations, imputations, or conversions of summary data were performed. Study characteristics, intervention features, psychological and microbiome-related outcomes, and risk-of-bias judgments were summarized in tables and figures to facilitate comparison across studies. No formal subgroup analyses or meta-regression were performed because of the qualitative nature of the synthesis and the substantial heterogeneity of the included studies. No sensitivity analyses were conducted because no quantitative meta-analysis was performed. Formal assessment of reporting bias, including funnel-plot asymmetry or statistical tests for small-study effects, was not performed because no quantitative meta-analysis was conducted and the included studies were highly heterogeneous with respect to interventions and outcomes. No formal assessment of the certainty of the body of evidence, such as GRADE, was undertaken.

The findings were initially organized according to the intervention categories represented among the eligible studies: psychobiotic interventions, dietary interventions, and other lifestyle-oriented interventions. In addition, a cross-intervention narrative synthesis was undertaken to identify recurrent microbiome-related patterns across the included RCTs. This synthesis examined repeated changes in specific bacterial taxa or taxonomic groups, global microbial diversity, SCFA-related features, and other microbiome-associated metabolites or functional characteristics. Particular attention was given to whether recurrent microbiome changes were observed across different intervention categories, whether they occurred in trials reporting favorable stress-related outcomes, and whether psychological and microbiome responses occurred in parallel. When interpreting recurrent taxonomic findings, changes involving bacterial groups directly administered as components of psychobiotic interventions were considered separately from changes in non-administered taxa or broader microbial functional features. Given the substantial heterogeneity in interventions, populations, microbiome methodologies, and outcome definitions, this cross-intervention synthesis was descriptive and hypothesis-generating and was not interpreted as evidence of mechanistic mediation. Although physical activity, sleep-related strategies, and mindfulness-based approaches were included within the search framework, no eligible standalone physical-activity or sleep-intervention RCTs met all predefined criteria requiring concurrent assessment of gut microbiome and stress-related outcomes. 

## 3. Results

### 3.1. Study Selection

The database search identified 424 records, including 76 from PubMed, 294 from Scopus, and 54 from the Cochrane Central Register of Controlled Trials (CENTRAL). After the removal of 188 duplicate records, 236 unique studies remained for title and abstract screening. Following the initial screening, 195 records were excluded because they did not meet the predefined eligibility criteria. The full texts of the remaining 41 articles were assessed for eligibility. Of these, 12 studies were excluded, including six that did not assess the gut microbiome, five with an ineligible study design, and one retracted publication. Ultimately, 29 randomized controlled trials met the eligibility criteria and were included in the qualitative synthesis. The complete study selection process is presented in Figure 1.

### 3.2. Characteristics of Included Studies

The characteristics of the included randomized controlled trials are presented in Table 2. A total of 29 RCTs published from 2016 through 26 April 2026 met the eligibility criteria and were included in the qualitative synthesis. Sample sizes ranged from 17 to 351 participants, while intervention duration ranged from 7 days to 24 weeks, with one study evaluating an intervention conducted across a substantial period of pregnancy.

The included studies evaluated a wide range of adult populations, including healthy adults, university students exposed to academic stress, individuals experiencing psychological stress or anxiety, older adults, menopausal women, pregnant women, and patients with gastrointestinal disorders, type 2 diabetes mellitus, or fatigue [63,64,65,66,67,68,69,70,71,72,73,74,75,76,77,78,79,80,81,82,83,84,85,86,87,88,89,90,91]. This substantial population heterogeneity limits direct comparability across trials, as baseline health status, metabolic characteristics, gastrointestinal conditions, and stress burden may influence both psychological and microbiome responses to intervention. Accordingly, cross-study findings were interpreted as patterns across heterogeneous populations rather than as directly comparable estimates of intervention effects.

Three broad categories of interventions were identified. The majority of studies (62.1%) investigated targeted psychobiotic interventions, primarily involving single- or multi-strain probiotic formulations. Thus, the available randomized evidence was unevenly distributed across intervention categories, with psychobiotic trials comprising the majority of included studies. Eight studies (27.6%) evaluated dietary interventions, including psychobiotic diets, high-fiber diets, fermented foods, functional foods, and specific bioactive dietary components. The remaining three studies (10.3%) evaluated broader lifestyle-oriented or personalized nutrition approaches, including a Mediterranean diet or mindfulness-based stress-reduction intervention during pregnancy, caloric restriction with or without adjunct psychological intervention, and a microbiome-based AI-assisted personalized nutrition intervention [89,90,91].

Across all intervention categories, psychological outcomes were assessed using validated questionnaires measuring perceived stress, anxiety, depressive symptoms, cognitive function, sleep quality, or quality of life. Gut microbiome outcomes primarily included changes in microbial composition, α-diversity, β-diversity, and the relative abundance of specific bacterial taxa, most commonly assessed using 16S rRNA gene sequencing. However, substantial methodological variability was present across studies in sequencing approaches, laboratory procedures, and bioinformatic pipelines, which limits direct comparison of microbiome findings across trials. Several studies additionally evaluated biological markers related to gut–brain axis function, including cortisol concentrations, inflammatory biomarkers, neurotransmitter-related metabolites, and microbial metabolic products [63,64,65,66,67,68,69,70,71,72,73,74,75,76,77,78,79,80,81,82,83,84,85,86,87,88,89,90,91].

Overall, the included RCTs represented a heterogeneous and unevenly distributed evidence base examining lifestyle-oriented and psychobiotic interventions targeting psychological stress and their potential effects on gut microbiome composition and function in adult populations.

### 3.3. Effects of Psychobiotic Interventions

Targeted psychobiotic interventions constituted the largest intervention category, representing 18 of the 29 included randomized controlled trials (62.1%). Most studies investigated single- or multi-strain probiotic formulations, predominantly belonging to the *Lactobacillus* and *Bifidobacterium* genera, whereas a smaller number evaluated fermented products or microbiome-targeted botanical preparations [63,64,65,66,67,68,69,70,71,72,73,74,75,76,77,78,79,80]. The predominance of psychobiotic trials resulted in an uneven distribution of evidence across intervention categories; consequently, findings from the overall evidence base are more strongly informed by probiotic interventions than by dietary, behavioral, or multimodal approaches.

Psychological and stress-related responses to psychobiotic interventions were favorable in several trials but were not uniform. Improvements were reported across perceived stress, anxiety, depressive symptoms, sleep-related outcomes, and selected cognitive measures, whereas other trials demonstrated null or outcome-specific effects [63,66,67,68,69,70,71,72,73,74,75,76,77,78,79,80]. Detailed study-level psychological and stress-related outcomes are presented in Table 2.

Microbiome responses to psychobiotic interventions were similarly heterogeneous. Several studies demonstrated preservation or attenuation of stress-associated changes in gut microbial diversity and composition [69,70,72], whereas others reported increases in specific bacterial taxa, including *Bifidobacterium*, *Faecalibacterium*, *Roseburia*, and members of the phylum Firmicutes [67,68,77,78,79]. However, significant global microbiome changes were not universal. Li J. et al. reported increased *Enterococcus faecalis* abundance and fecal tryptamine without significant overall changes in microbiota composition or diversity [65], while Bloemendaal et al. found no significant intervention effect on overall β-diversity or individual taxa after multiple-comparison correction, despite an association between Ruminococcaceae_UCG-003 and protection of working memory under stress [73]. These findings indicate that psychobiotic effects may involve selective taxonomic or functional changes rather than uniform shifts in global microbial diversity.

Several RCTs additionally evaluated biological markers associated with gut–brain axis function. Reductions in cortisol or other HPA-axis-related markers were reported following selected psychobiotic interventions [63,64,70], while Nishida et al. observed reduced salivary chromogranin A [69]. Increased GABA levels were also reported following administration of *Weizmannia coagulans* BC99 [68]. These findings suggest that selected psychobiotic interventions may influence neuroendocrine or neuroactive pathways related to stress regulation, although such effects were not consistently accompanied by significant improvements in psychological outcomes.

Despite the favorable pattern of findings, substantial heterogeneity was observed across probiotic strains, formulations, intervention durations, participant populations, microbiome methodologies, and outcome measures. Consequently, quantitative comparisons remain challenging. Overall, the evidence suggests that psychobiotic interventions may influence psychological, neuroendocrine, and microbiome-related outcomes, although these effects were neither universal nor consistently observed in parallel within trials.

### 3.4. Effects of Dietary Interventions

Dietary interventions were evaluated in eight RCTs (27.6%), encompassing a broad spectrum of nutritional approaches, including psychobiotic diets, high-fiber dietary patterns, fermented foods, functional foods, and supplementation with specific bioactive compounds [81,82,83,84,85,86,87,88].

Psychological and stress-related responses to dietary interventions were heterogeneous. Favorable effects on perceived stress, mood, anxiety, depressive symptoms, or related stress markers were reported in selected trials [81,82,83,86,88], whereas other dietary interventions demonstrated null or outcome-specific psychological effects [84,85,87]. Detailed study-level psychological and stress-related outcomes are presented in Table 2.

Gut microbiome effects were likewise heterogeneous. Walnut supplementation attenuated stress-associated reductions in microbial diversity in females [82], while the high-fiber intervention increased microbial richness and diversity and altered several bacterial taxa [83]. Ginger supplementation modified specific taxa without significantly altering α- or β-diversity [84], whereas blackcurrant supplementation produced no significant overall effects on microbial diversity, composition, or functional potential despite baseline *Bifidobacterium* abundance being associated with working-memory response [85]. Oligofructose and combined oligofructose/2′-fucosyllactose interventions increased several taxa, including *Bifidobacterium*, *Roseburia*, and *Faecalibacterium prausnitzii* [86], while fermented dairy consumption significantly influenced β-diversity without broader significant microbiome changes [87]. Lignan supplementation was associated with enrichment of specific gut bacteria, particularly *Bacteroides ovatus*, and changes in lignan-derived microbial metabolites [88].

Taken together, dietary interventions may influence psychological, inflammatory, neuroendocrine, microbial, and metabolic components of the microbiota–gut–brain axis; however, improvements in psychological and microbiome outcomes did not consistently occur in parallel. The marked heterogeneity in dietary exposures, study populations, intervention duration, and outcome assessment precludes conclusions regarding a uniform dietary effect and highlights the need for larger and more standardized randomized trials.

### 3.5. Effects of Other Lifestyle-Oriented and Personalized Nutrition Interventions

Three additional RCTs evaluated broader lifestyle-oriented or personalized nutrition approaches that did not fit exclusively within the main psychobiotic or conventional dietary categories [89,90,91]. These included a pregnancy trial comparing Mediterranean diet or MBSR with usual care [89], a caloric-restriction intervention with or without adjunct clinical psychological intervention [90], and a microbiome-based AI-assisted personalized nutrition intervention compared with a low-FODMAP diet in patients with irritable bowel syndrome [91].

Microbiome responses differed substantially across these three interventions. In the IMPACT BCN microbiome subanalysis, the Mediterranean diet significantly influenced overall gut microbiota structure and increased several Lachnospiraceae, Ruminococcaceae, and other SCFA-producing taxa, whereas microbiome effects associated with MBSR were more limited [89]. Caloric restriction was associated with longitudinal changes in α- and β-diversity, while the adjunct clinical psychological intervention exerted little additional effect on the microbiome [90]. In patients with irritable bowel syndrome, the microbiome-based AI-assisted personalized dietary intervention induced significant shifts in microbial diversity and taxonomic composition compared with the low-FODMAP diet [91]. Psychological and clinical outcomes varied across these heterogeneous interventions and are detailed in Table 2.

Collectively, these studies illustrate the potential of broader lifestyle and personalized nutrition strategies to influence psychological, clinical, and microbiome-related outcomes. However, their substantially different designs and intervention components prevent direct comparison and do not establish the superiority of integrated or multimodal approaches.

A summary of the principal findings across the three intervention categories, including psychobiotic, dietary, and other lifestyle-oriented interventions, is presented in Table 3.

### 3.6. Shared Microbiome Patterns Across Intervention Categories

Across intervention categories, no single taxonomic or diversity pattern consistently characterized trials reporting favorable stress-related outcomes. Nevertheless, several recurrent microbiome-related signals were identified. Increases in *Bifidobacterium* or Bifidobacteriaceae were reported across psychobiotic and dietary interventions [67,74,78,83,86], while increases in *Lactobacillus* or Lactobacillaceae were reported in several psychobiotic and dietary trials [67,74,83]. *Faecalibacterium* or *Faecalibacterium prausnitzii* increases were observed in both psychobiotic and dietary interventions [68,86], and increases in *Roseburia* were reported in psychobiotic and dietary trials [78,86]. Changes involving Ruminococcaceae- or Lachnospiraceae-related taxa were additionally reported across different intervention approaches [72,73,89]. These findings indicate some recurrence of specific bacterial groups across otherwise heterogeneous interventions, although none of these taxonomic changes were consistently observed across all trials reporting favorable stress-related outcomes.

Functional and diversity-related findings were similarly heterogeneous. Several trials reported changes in SCFA concentrations, SCFA-producing taxa, or related microbial features [63,64,68,74,89], while other studies identified changes involving tryptophan-related microbial metabolites [65,81]. However, improvements in psychological or stress-related outcomes could occur despite limited or non-significant changes in global microbial diversity, as observed in selected trials [66,73,85]. Conversely, microbiome alterations were also reported in studies without significant improvement in the principal psychological outcomes [65,84,87]. Taken together, the available RCTs indicate recurrent taxonomic and functional signals rather than a uniform microbiome signature associated with successful stress-targeting interventions. Psychological and microbiome responses therefore did not invariably occur in parallel, and the heterogeneity of the findings precludes identification of a single microbial pattern common to all effective interventions.

### 3.7. Risk of Bias Assessment

**Figure 2:**
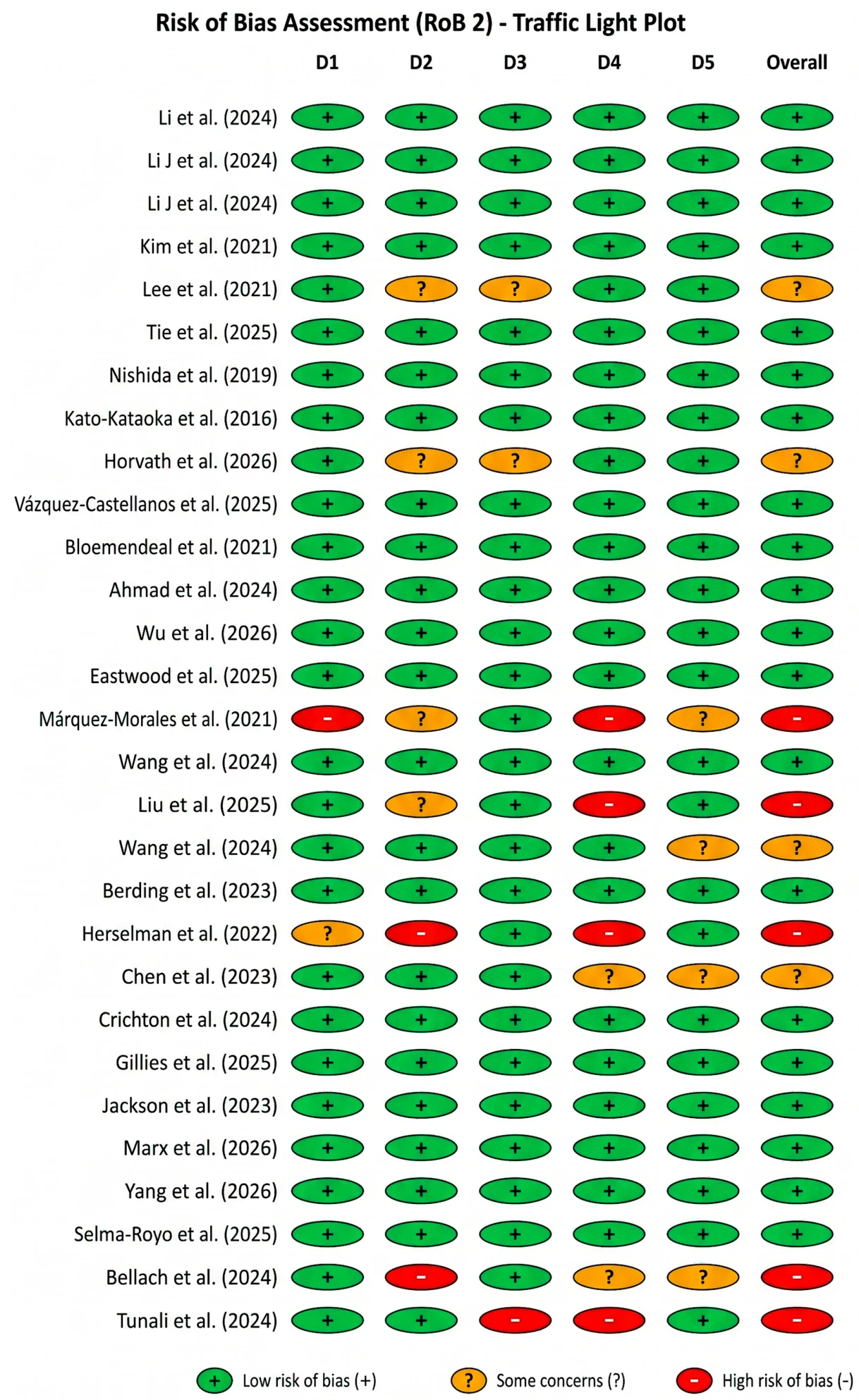
Risk-of-bias assessment using the RoB 2 tool for RCTs—Traffic Light Plot [63,64,65,66,67,68,69,70,71,72,73,74,75,76,77,78,79,80,81,82,83,84,85,86,87,88,89,90,91].

The methodological quality of the included RCTs was assessed using the Cochrane Risk of Bias 2 (RoB 2) tool. Overall, 20 of the 29 studies (69.0%) were judged to be at low risk of bias, four (13.8%) raised some concerns, and five (17.2%) were judged to be at high risk of bias. The studies rated as having some concerns were Lee et al. (2021) [67], Horvath et al. (2026) [71], Wang et al. (2024) [80], and Chen et al. (2023) [83], whereas Márquez-Morales et al. (2021) [77], Liu et al. (2025) [79], Herselman et al. (2022) [82], Bellach et al. (2024) [90], and Tunali et al. (2024) [91] were judged to be at high risk of bias. The identified concerns involved different RoB 2 domains, including deviations from intended interventions (D2), missing outcome data (D3), measurement of the outcome (D4), and selection of the reported result (D5), with concerns related to the randomization process (D1) identified in selected studies. Findings from studies judged to be at high risk of bias were interpreted cautiously in the overall synthesis. Detailed domain-level judgments are presented in Table 4 and Figure 2.

## 4. Discussion

### 4.1. Principal Findings

The present systematic review synthesized evidence from 29 RCTs evaluating the effects of lifestyle-oriented and psychobiotic interventions on psychological stress and gut microbiome-related outcomes in adults. Overall, the findings indicate that lifestyle interventions targeting the gut–brain axis may influence both psychological and biological outcomes, although the magnitude, direction, and consistency of these effects varied according to intervention type, study population, intervention duration, and microbiome assessment methodology. Among these sources of heterogeneity, differences in intervention characteristics and microbiome assessment methodology appear particularly important for interpreting the inconsistent findings. Psychobiotic trials differed substantially in probiotic strains, formulations, doses, and intervention durations, while dietary and behavioral interventions varied in their components and intensity. In parallel, differences in sequencing approaches, laboratory procedures, and bioinformatic pipelines may have contributed to variation in the microbial taxa and diversity measures detected across studies. Population characteristics and baseline health status represent additional sources of variability; however, the available evidence does not permit the relative contribution of these factors to be formally quantified.

A principal finding of this review is that favorable stress-related outcomes were not associated with a single, reproducible microbiome signature across the included RCTs. Instead, several recurrent taxonomic and functional signals emerged across otherwise heterogeneous interventions. Increases in *Bifidobacterium* or Bifidobacteriaceae were reported across psychobiotic and dietary interventions [67,74,78,83,86], while increases in *Lactobacillus* or Lactobacillaceae were also observed in several trials [67,74,83]. Recurrent changes involving *Faecalibacterium*, *Roseburia*, and members of the Lachnospiraceae/Ruminococcaceae families were identified across different intervention approaches [68,72,73,78,86,89]. At the functional level, several studies reported changes in SCFA concentrations, SCFA-producing taxa, or related microbial features [63,64,68,74,89], while others identified alterations in tryptophan-related microbial metabolites [65,81]. Importantly, these microbiome-related changes were not consistently observed across all trials reporting favorable stress-related outcomes. Psychological improvements could occur despite limited or non-significant changes in global microbial diversity [66,73,85], whereas microbiome alterations were also reported in the absence of significant improvement in the principal psychological outcomes [65,84,87]. Collectively, these findings indicate partial taxonomic and functional convergence rather than a uniform microbial response and do not establish that microbiome alterations mechanistically mediated the observed psychological effects. Moreover, when psychological and microbiome-related changes occurred concurrently, their co-occurrence alone cannot establish a causal relationship, as parallel responses may reflect independent effects of the intervention or non-specific influences, including placebo-related effects or regression to the mean.

An important consideration when interpreting this apparent cross-study convergence is that increases in *Lactobacillus*- and *Bifidobacterium*-related taxa cannot necessarily be regarded as independent evidence of a shared mechanistic microbiome response. In several psychobiotic trials, strains belonging to these bacterial groups were themselves components of the administered intervention; for example, supplementation with *Lactobacillus reuteri* and *Bifidobacterium adolescentis* was accompanied by increases in Lactobacillaceae and Bifidobacteriaceae [67], while *Bifidobacterium breve* supplementation was associated with increased *Bifidobacterium* abundance [78]. Such findings may therefore partly reflect direct supplementation rather than an endogenous microbial response associated with improvement in stress-related outcomes. Greater mechanistic relevance may lie in secondary changes extending beyond the supplemented organisms, including alterations in non-administered taxa, microbial diversity, SCFA-related features, and other microbial metabolites or functional pathways [63,64,68,69,72,74,78]. However, these secondary microbiome responses were heterogeneous across the included RCTs and did not define a reproducible cross-intervention signature.

Among the different intervention categories, psychobiotic supplementation represented the largest body of evidence, accounting for more than half of the included studies. This imbalance should be considered when interpreting the overall synthesis, because recurrent microbiome patterns identified across the review are disproportionately informed by psychobiotic trials and may therefore be less representative of dietary, behavioral, or multimodal interventions. Across diverse populations—including healthy adults, university students exposed to academic stress, older adults, menopausal women, and patients with gastrointestinal disorders—probiotic supplementation was frequently, although not uniformly, associated with improvements in perceived stress, anxiety, sleep quality, or neuroendocrine outcomes [63,67,69,71,74,75]. These findings indicate that favorable stress-related responses to psychobiotic interventions have been observed across different physiological and clinical contexts, although important differences between probiotic strains, formulations, and study populations should be acknowledged. Although the parallel psychological and microbiological changes observed in several trials do not establish a causal or mediating role of the microbiome, when considered alongside the broader mechanistic and therapeutic literature, they support the biological plausibility and therapeutic potential of targeting the gut–brain axis [92,93].

The recurrence of selected microbial taxa and functional features across otherwise heterogeneous interventions may be biologically relevant even in the absence of consistent changes in global microbial diversity. In particular, several of the bacterial groups identified across the included trials are associated with SCFA production and with microbial functions involved in intestinal barrier integrity, immune homeostasis, and gut–brain communication [94,95,96]. Accordingly, the cross-study patterns identified in this review may reflect functional adaptations within the gut microbiome that are not fully captured by global diversity indices. However, given the heterogeneity of the observed microbial responses and the absence of consistent parallel changes in psychological and microbiome outcomes, these findings should be interpreted as biologically plausible signals rather than evidence of a common mechanistic pathway.

Dietary interventions produced heterogeneous findings across both psychological and microbiome-related outcomes. Whole-diet approaches, including psychobiotic and Mediterranean-style dietary interventions, showed potentially favorable effects on stress-related and microbiome outcomes in selected trials [81,89], whereas fiber-rich diets and interventions based on individual foods or bioactive components produced more variable and outcome-specific responses [82,83,84,85,86,87,88]. These findings suggest that the effects of dietary interventions on gut–brain communication may depend substantially on dietary composition, population characteristics, and the outcomes assessed, as well as that overall dietary quality may exert a greater influence on gut–brain communication than isolated nutrients alone [97,98].

Three studies evaluated broader lifestyle-oriented or personalized approaches beyond the main psychobiotic and dietary categories [89,90,91]. These included Mediterranean dietary or mindfulness-based intervention during pregnancy [89], caloric restriction with or without adjunct psychological intervention [90], and microbiome-based AI-assisted personalized nutrition [91]. Given their substantially different designs, populations, and therapeutic components, these studies should not be interpreted as a homogeneous multimodal intervention category. Nevertheless, their findings illustrate the potential for broader lifestyle-oriented approaches to influence psychological, clinical, and microbiome-related outcomes through multiple pathways. This possibility is consistent with the broader concept that combined lifestyle modification may act through complementary biological mechanisms along the gut–brain axis [99,100].

Despite these encouraging observations, the review also revealed substantial methodological heterogeneity across studies. Considerable variation existed regarding participant characteristics, intervention protocols, follow-up duration, microbiome sequencing techniques, outcome measures, and analytical approaches. Furthermore, the lack of consistent parallel changes in psychological outcomes and global microbial diversity suggests that broad diversity metrics alone may not adequately capture microbiome-related responses to stress-targeting interventions. Functional characteristics, including microbial metabolic activity, production of bioactive metabolites, and changes in selected bacterial taxa, may therefore provide complementary information beyond global diversity measures [101,102].

### 4.2. Biological Interpretation of the Gut–Brain Axis

The findings of the present review support the growing body of evidence suggesting that the gut microbiome constitutes a dynamic biological interface linking lifestyle behaviors with psychological stress regulation [103]. Although the included interventions differed considerably in their design, intensity, and duration, a pattern emerged in several trials in which improvements in psychological outcomes coincided with changes in gut microbial composition or function. This co-occurrence is consistent with the gut–brain axis as a biologically plausible framework linking lifestyle interventions, microbial responses, and psychological outcomes [104,105,106]; however, the included RCTs do not establish that the observed microbiome changes mediated the psychological effects.

One of the most plausible biological pathways underlying these observations involves modulation of the HPA axis. Chronic psychological stress promotes sustained activation of the HPA axis, leading to prolonged cortisol secretion, increased intestinal permeability, immune dysregulation, and alterations in gut microbial composition [107]. Several randomized trials included in this review demonstrated reductions in cortisol concentrations following psychobiotic supplementation, accompanied by improvements in perceived stress or sleep quality [63,64,69]. Although these parallel neuroendocrine and microbiome-related observations are compatible with biological interaction along the gut–brain axis, the available trials do not establish whether microbiome modulation contributed causally to the observed changes in stress-related neuroendocrine activity.

Beyond changes involving directly supplemented organisms, several trials identified secondary taxonomic or functional responses that may be relevant to gut–brain communication, including changes in *Faecalibacterium*, *Roseburia*, Ruminococcaceae/Lachnospiraceae-related taxa, SCFA-producing bacteria, and SCFA-related microbial features [68,72,74,78,86,89]. SCFAs, particularly butyrate, acetate, and propionate, have established roles in intestinal barrier integrity, immune regulation, and neuroendocrine signaling [108]. These observations provide biological plausibility for functional microbiome involvement but should not be interpreted as demonstrating that such microbial changes mediated the psychological effects observed in the included trials.

Interestingly, improvements in psychological outcomes were not always accompanied by significant changes in global microbial diversity. Psychological and microbiome-related responses did not consistently occur in parallel. Some interventions produced favorable psychological or cognitive outcomes despite limited or non-significant global microbiome changes [66,73,85], whereas other trials identified microbiome-related alterations without significant improvement in the principal psychological outcomes [65,84,87]. These findings suggest that overall diversity alone may not adequately capture biologically relevant changes within the gut microbiome. Instead, functional characteristics—including microbial metabolic capacity, abundance of specific bacterial taxa, and production of bioactive metabolites—may represent more sensitive indicators of gut–brain axis modulation [109]. This observation also highlights the limitations of relying exclusively on diversity metrics when evaluating microbiome-targeted interventions.

Neuroactive signaling provides another potential mechanism linking microbial modulation with improvements in psychological health. Several studies included in this review suggested that psychobiotic interventions may influence pathways related to GABA, serotonin metabolism, and other neuroactive compounds involved in emotional regulation. For example, supplementation with *Weizmannia coagulans* was associated with increased circulating GABA concentrations together with reductions in anxiety symptoms [68], whereas *Bifidobacterium breve* supplementation was linked to improvements in sleep quality and reductions in cortisol [64]. Furthermore, dietary interventions involving fermented foods were associated with neurochemical alterations affecting hippocampal metabolism [87]. Although the precise causal pathways remain incompletely understood, these findings support the hypothesis that microbial metabolites may influence central nervous system function through multiple interacting neural, endocrine, and metabolic pathways [110,111].

The findings from the broader lifestyle-oriented interventions further illustrate the biological complexity of the gut–brain axis. Although the interventions evaluated in these trials were heterogeneous and should not be considered a single therapeutic category [89,90,91], they highlight the potential relevance of simultaneously addressing nutritional, psychological, metabolic, and behavioral determinants of health. This observation is consistent with a growing body of evidence indicating that comprehensive behavioral lifestyle interventions may exert broad psychophysiological effects across diverse clinical populations. For example, the Pythagorean Self-Awareness Intervention (PSAI), a structured cognitive–behavioral lifestyle intervention that integrates stress management, self-reflection, and behavioral modification, has demonstrated beneficial effects in patients with major depressive disorder and irritable bowel syndrome [112,113]. Similarly, broader lifestyle interventions combining behavioral counseling with dietary modification have been associated with concurrent improvements in psychological well-being, lifestyle behaviors, and biological markers related to oxidative stress and healthy aging [114]. Such approaches may therefore produce complementary effects across multiple interconnected pathways, including reduced HPA axis activation, improved vagal signaling, enhanced intestinal barrier function, and attenuation of chronic low-grade inflammation [115,116]. Collectively, these findings support the concept that the gut–brain axis should be viewed as an integrated biological network rather than a collection of isolated mechanisms.

**Figure 3:**
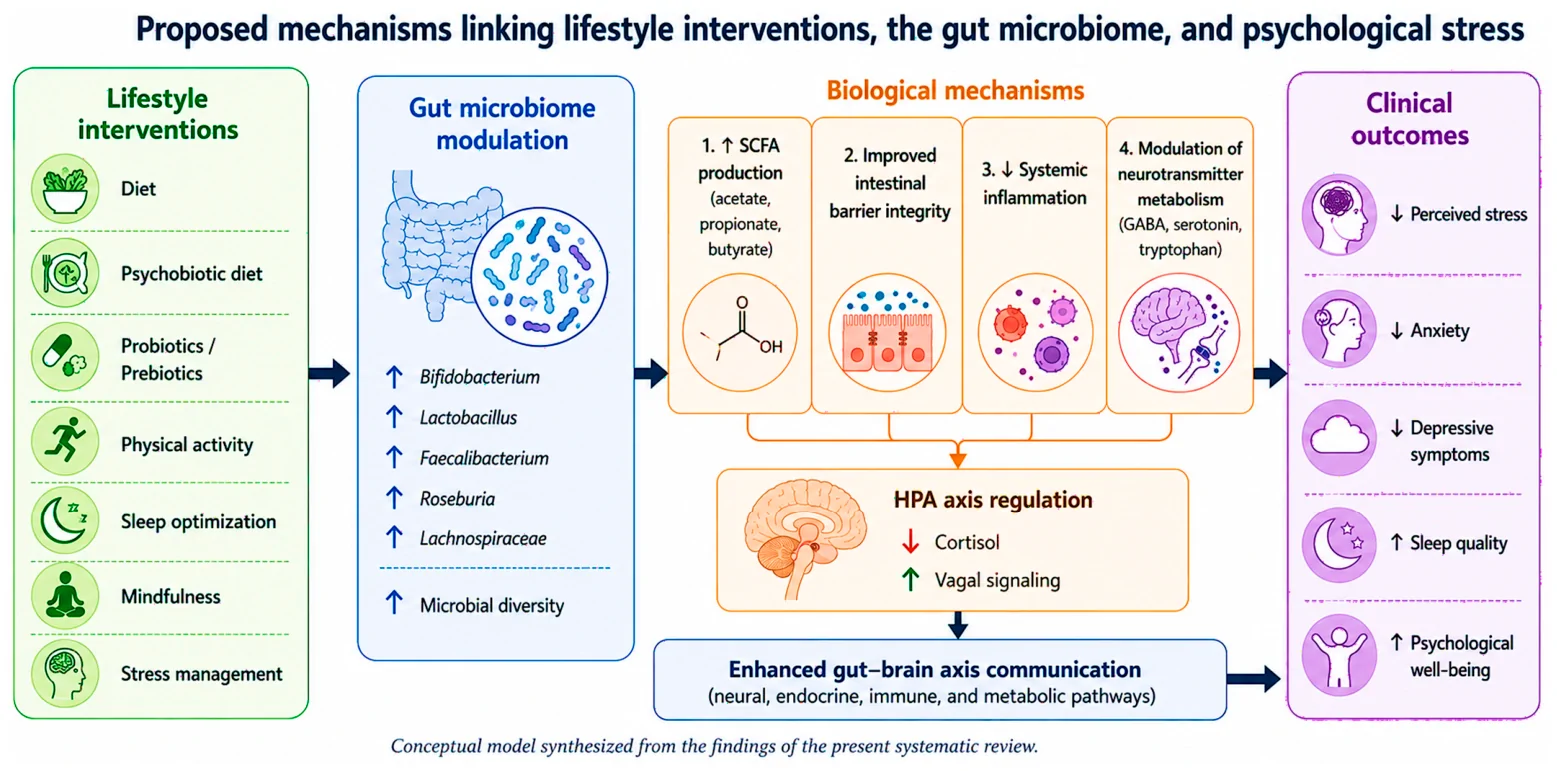
Conceptual framework of potential biological pathways linking lifestyle interventions, gut microbiome responses, and psychological stress. Potential pathways include changes in microbial composition and function, short-chain fatty acid production, intestinal barrier integrity, systemic inflammation, hypothalamic–pituitary–adrenal axis activity, neurotransmitter metabolism, and gut–brain communication. The framework integrates findings from the included RCTs with biologically plausible mechanisms discussed in the broader gut–brain axis literature and should not be interpreted as evidence that microbiome changes mediated the psychological effects observed in the included trials. The framework was developed narratively rather than through quantitative or mediation analysis, and the depicted links represent proposed conceptual relationships rather than established causal pathways. Arrows indicate the direction of change: ↑, increase; ↓, decrease.

Taken together, the evidence synthesized in this review is consistent with multiple biological pathways potentially linking lifestyle interventions, gut microbiome responses, and stress regulation, including neuroendocrine signaling, microbial metabolism, immune function, and gut barrier integrity. However, the substantial heterogeneity of microbiome responses, together with the inconsistent parallel occurrence of psychological and microbial changes, precludes attribution of the observed stress-related effects to a specific microbiome-mediated mechanism. The pathways discussed above should therefore be regarded as biologically plausible mechanisms supported by the broader gut–brain axis literature rather than as mediation pathways established by the included RCTs. The principal biological pathways considered in relation to the findings of the present systematic review are summarized conceptually in Figure 3.

### 4.3. Clinical Implications

The findings of the present systematic review have several clinically relevant implications for the prevention and management of stress-related disorders. Collectively, the available evidence suggests that lifestyle interventions targeting the gut–brain axis may represent valuable adjunctive strategies for improving psychological well-being while simultaneously promoting gastrointestinal and metabolic health. Rather than acting as isolated therapeutic approaches, dietary modification, psychobiotic supplementation, and broader lifestyle interventions appear to influence multiple physiological systems involved in the stress response, supporting a more integrated model of patient care [117,118].

Among the available interventions, psychobiotic supplementation represented the largest and comparatively most consistent body of evidence, with favorable effects reported across a wide range of populations, including healthy individuals, students experiencing academic stress, older adults, menopausal women, and patients with gastrointestinal disorders [63,67,69,71,74,75]. However, the considerable variability in probiotic strains, formulations, dosages, and treatment duration observed across studies indicates that probiotic supplementation should not yet be considered a uniform therapeutic approach. Instead, future clinical recommendations will likely require greater emphasis on strain-specific efficacy and individualized treatment selection.

The present findings also support the growing concept that dietary patterns may exert broader and potentially more sustainable effects than isolated nutritional supplements. Whole dietary approaches, including psychobiotic and Mediterranean-style diets, demonstrated potentially favorable effects on stress-related and gut microbiome outcomes in selected trials [81,89]. These observations reinforce current recommendations emphasizing overall dietary quality rather than individual nutrients as a cornerstone of lifestyle medicine. Furthermore, dietary interventions may represent practical and accessible strategies with potential for integration into routine clinical care, although their long-term feasibility and effectiveness require further investigation.

An additional clinically relevant observation concerns the broader lifestyle-oriented interventions evaluated in a small subset of trials. Although only three RCTs examined approaches beyond the main psychobiotic and dietary categories, these studies reported potentially relevant changes across psychological, clinical, and microbiome-related outcomes [89,90,91]. However, their substantially different intervention designs preclude conclusions regarding the comparative effectiveness or potential superiority of integrated or multimodal lifestyle programs. Further large-scale randomized trials directly comparing single-component and combined interventions are required before firm clinical recommendations can be established.

Despite these encouraging findings, the current evidence should be interpreted with appropriate caution. Considerable heterogeneity existed regarding participant characteristics, baseline health status, microbiome sequencing methodologies, intervention protocols, and outcome assessment. Moreover, relatively short intervention periods characterized many of the included studies, limiting conclusions regarding the long-term sustainability of the observed microbiome and psychological changes. Consequently, the available evidence supports further evaluation of lifestyle interventions as potential complementary, rather than replacement, strategies within established clinical management pathways.

Taken together, the findings of this review support a growing shift toward more holistic and personalized approaches to stress management. The convergence of nutritional science, microbiome research, behavioral medicine, and lifestyle medicine provides an opportunity to develop interventions that address both the biological and psychological dimensions of stress. As the evidence base continues to expand, modulation of the gut–brain axis may become an increasingly important component of precision lifestyle medicine, offering novel opportunities for the prevention and management of stress-related disorders [119,120].

### 4.4. Strengths and Limitations

The present systematic review possesses several important strengths that enhance the reliability and clinical relevance of its findings. First, to our knowledge, this is among the most up-to-date systematic reviews focusing specifically on the effects of lifestyle-oriented and psychobiotic interventions on both psychological stress and the gut microbiome in adults, incorporating evidence from RCTs published through April 2026. By restricting inclusion to RCTs, the present review provides a higher level of evidence than previous reviews that combined observational studies, uncontrolled interventions, and preclinical research. A second major strength lies in the evaluation of multiple intervention categories within a single evidence synthesis. Rather than focusing exclusively on probiotics or dietary supplementation, this review simultaneously evaluated psychobiotic supplementation, dietary interventions, and other lifestyle-oriented approaches, thereby providing a broader understanding of how different intervention strategies may influence the gut–brain axis. This integrated perspective reflects the multifactorial nature of psychological stress and recognizes that gut microbiome modulation is unlikely to depend on a single therapeutic approach. Another strength is the simultaneous consideration of psychological, neuroendocrine, and microbiome-related outcomes. Most included studies evaluated both clinical and biological endpoints, allowing the present review to examine whether improvements in perceived stress were accompanied by measurable changes in gut microbial composition or function. This dual perspective provides greater biological plausibility for the observed associations than would be possible through assessment of psychological outcomes alone. The methodological quality of the included evidence was variable. Risk-of-bias assessment using the Cochrane RoB 2 tool indicated that 20 of the 29 RCTs (69.0%) were judged to have a low overall risk of bias, four studies were classified as presenting some concerns, and five studies were judged to have a high overall risk of bias. Accordingly, findings from studies with methodological limitations were interpreted cautiously, and risk-of-bias judgments should be considered separately from the overall certainty of the evidence.

Nevertheless, several limitations should be acknowledged when interpreting the results. Considerable clinical and methodological heterogeneity existed across the included studies. Participants differed substantially with respect to age, sex, baseline health status, psychological characteristics, and underlying medical conditions, while interventions varied in duration, intensity, probiotic strains, dietary protocols, and behavioral components. Such population heterogeneity is particularly relevant to the interpretation of microbiome outcomes, because baseline health status, metabolic characteristics, gastrointestinal conditions, and stress burden may influence both baseline microbial profiles and responsiveness to intervention. Consequently, differences in microbiome responses across trials cannot be attributed solely to intervention type, and comparisons between clinically distinct populations should be interpreted cautiously. The evidence base was also imbalanced across intervention categories, with psychobiotic trials accounting for 62.1% of included RCTs; consequently, the overall synthesis is more strongly informed by psychobiotic interventions than by dietary, behavioral, or multimodal approaches. Similarly, microbiome analyses were performed using different sequencing platforms, laboratory protocols, and bioinformatic pipelines, limiting direct comparisons across studies. A further limitation concerns the diversity of outcome measures used to assess both psychological stress and gut microbiome characteristics. Although validated psychometric instruments were generally employed, studies differed in the scales used to evaluate stress, anxiety, depressive symptoms, sleep quality, and quality of life. Likewise, microbiome outcomes ranged from taxonomic composition and diversity indices to specific bacterial taxa and microbial metabolites, reducing opportunities for direct quantitative comparison and precluding formal meta-analysis. The narrative nature of the synthesis also limits the ability to estimate pooled effect sizes or formally quantify between-study heterogeneity, and the direction and statistical significance of findings should therefore not be interpreted as evidence of comparable effect magnitude across interventions. The relatively short duration of many interventions also limits conclusions regarding the long-term sustainability of the observed effects. Most randomized trials lasted between four and twelve weeks, making it difficult to determine whether improvements in psychological outcomes and gut microbiome composition persist following discontinuation of the intervention. Long-term follow-up studies remain scarce and represent an important area for future investigation. Finally, although this review focused exclusively on RCTs to maximize methodological rigor, this approach inevitably excluded potentially informative observational evidence and mechanistic studies that may provide additional insight into causal pathways linking lifestyle behaviors, psychological stress, and the gut microbiome. Consequently, while the present findings offer a robust summary of current clinical evidence, they should be interpreted within the context of the available randomized literature rather than the broader microbiome field. In addition, the restriction to English-language publications may have resulted in the exclusion of relevant evidence published in other languages. Finally, the review protocol was not prospectively registered, which should be considered when interpreting the methodological transparency of the review.

Despite these limitations, the exclusive inclusion of RCTs and the simultaneous evaluation of psychological and microbiome-related outcomes provide a focused synthesis of the available randomized evidence. Nevertheless, the methodological heterogeneity identified across studies highlights the need for greater standardization of future clinical trials to facilitate more precise comparisons and stronger evidence synthesis.

### 4.5. Future Directions

Despite the encouraging findings identified in this systematic review, several important knowledge gaps remain and should be addressed by future research. One of the major priorities is the standardization of study design, including participant selection, intervention protocols, microbiome assessment methods, and outcome measures. The substantial methodological heterogeneity observed across the included RCTs limits direct comparisons between studies and hinders the development of evidence-based clinical recommendations. Greater harmonization of microbiome sequencing methodologies, bioinformatic pipelines, and psychological outcome assessment would substantially improve the comparability and reproducibility of future investigations.

Future RCTs should also incorporate longer intervention and follow-up periods. Most studies included in the present review evaluated interventions lasting between four and twelve weeks, providing limited information regarding the long-term stability of microbiome changes or the persistence of psychological improvements. Extended follow-up assessments are necessary to determine whether the beneficial effects observed during active intervention are maintained after treatment discontinuation and whether repeated or maintenance interventions are required to sustain clinical benefits.

Another important research priority is the identification of the biological mechanisms responsible for individual variability in treatment response. The considerable heterogeneity observed across participants suggests that baseline microbiome composition, dietary habits, metabolic status, genetic background, medication use, and environmental exposures may all influence responsiveness to lifestyle and psychobiotic interventions. Future studies integrating microbiome sequencing with metabolomics, transcriptomics, proteomics, and other multi-omics approaches may provide a more comprehensive understanding of the biological pathways linking the gut microbiome with psychological stress.

Further investigation is also warranted regarding the comparative effectiveness of different intervention strategies. Although broader lifestyle-oriented and multimodal approaches are theoretically well suited to target multiple components of the gut–brain axis simultaneously, the present review does not provide sufficient comparative evidence to determine whether they offer greater benefits than single-component interventions. Direct head-to-head comparisons between psychobiotic supplementation, dietary interventions, physical activity, mindfulness-based programs, and integrated lifestyle approaches would help determine the most effective therapeutic strategies for different populations.

Finally, future research should move beyond taxonomic characterization of the gut microbiome and place greater emphasis on microbial function. Functional outcomes, including short-chain fatty acid production, neurotransmitter metabolism, microbial gene expression, intestinal permeability, inflammatory pathways, and neuroendocrine biomarkers, may provide more clinically meaningful insights into gut–brain communication than changes in microbial diversity alone. The integration of these biological markers with validated psychological outcomes could substantially strengthen future evidence regarding the mechanisms through which lifestyle interventions influence stress regulation.

Collectively, these research priorities may contribute to the development of more standardized, personalized, and mechanism-based interventions targeting the gut–brain axis. As methodological consistency improves and high-quality randomized evidence continues to accumulate, future studies will be better positioned to translate microbiome research into practical clinical applications for the prevention and management of stress-related disorders.

## 5. Conclusions

This systematic review synthesized evidence from 29 RCTs investigating the effects of lifestyle-oriented and psychobiotic interventions on psychological stress and the gut microbiome in adults. Across intervention categories, no single taxonomic or diversity signature consistently characterized trials reporting favorable stress-related outcomes. Nevertheless, recurrent changes involving selected bacterial taxa, including *Bifidobacterium*, *Lactobacillus*, *Faecalibacterium*, *Roseburia*, and members of the Lachnospiraceae/Ruminococcaceae families, together with SCFA-related microbial features, were identified across several interventions. These findings suggest partial taxonomic and functional convergence rather than a uniform microbiome response. Importantly, psychological and microbiome-related changes did not consistently occur in parallel, and the available randomized evidence does not establish that microbiome alterations mediated the observed improvements in stress-related outcomes.

Among the evaluated interventions, psychobiotic supplementation represented the largest body of evidence and demonstrated the most consistent improvements in stress-related outcomes across diverse populations. Dietary interventions, particularly whole dietary patterns such as psychobiotic and Mediterranean-style diets, were associated with broader effects on microbial diversity and metabolic pathways relevant to gut–brain communication. Evidence regarding broader lifestyle-oriented and personalized approaches remains limited and heterogeneous, precluding conclusions regarding whether integrated or multimodal interventions provide greater benefits than single-component approaches.

Despite these encouraging findings, important methodological challenges remain. Considerable heterogeneity in study populations, intervention characteristics, microbiome assessment techniques, and outcome measures limits direct comparisons across studies and precludes definitive conclusions regarding the superiority of specific intervention strategies. Furthermore, the relatively short duration of most randomized trials highlights the need for longer-term investigations evaluating the sustainability of both psychological and microbiome-related effects.

Overall, the findings highlight the gut microbiome as a potentially relevant component of the biological response to lifestyle-oriented and psychobiotic interventions, while demonstrating substantial heterogeneity in the microbial changes associated with favorable stress-related outcomes. Future well-designed RCTs incorporating standardized microbiome methodologies, longer follow-up periods, and functional microbial analyses are needed to determine whether the recurrent taxonomic and metabolic signals identified in this review are reproducible and whether they contribute mechanistically to improvements in stress-related outcomes.

## Figures and Tables

**Table 1:** Eligibility criteria according to the PICO framework.

PICO Component	Eligibility Criteria
Population (P)	Adults (≥18 years), irrespective of sex or health status, in whom psychological stress or closely related psychological outcomes were assessed using validated psychometric instruments and/or stress-responsive biological markers such as cortisol.
Intervention (I)	Lifestyle-oriented and psychobiotic interventions potentially capable of modifying psychological stress and the gut microbiome, including dietary interventions or dietary patterns (e.g., Mediterranean diet), physical activity, mindfulness-based interventions, sleep-related interventions, and targeted probiotic or prebiotic supplementation.
Comparator (C)	Placebo, no-intervention control, usual care, or another appropriate comparator. Baseline measurements were also considered where appropriate for interpreting intervention effects.
Outcomes (O)	Eligible randomized trials were required to provide at least one gut microbiome outcome together with at least one stress-related psychological or biological outcome, either within the same publication or across clearly linked reports arising from the same randomized trial. Eligible stress-related outcomes included direct measures of perceived or psychological stress, stress-responsive neuroendocrine biomarkers such as cortisol, and psychological or behavioral outcomes closely associated with stress regulation, including anxiety, depressive symptoms, and sleep-related outcomes. Inflammatory biomarkers and neuroactive compounds were considered complementary biological outcomes only when reported within studies that also assessed a psychological or neuroendocrine stress-related endpoint. Eligible microbiome outcomes included microbial composition, α-diversity, β-diversity, relative abundance of bacterial taxa, and microbiome-related functional measures. Gut microbiome analyses performed using 16S rRNA gene sequencing, shotgun metagenomic sequencing, or equivalent molecular techniques were considered eligible.

**Table 2:** Characteristics of the Included Randomized Controlled Trials and Their Main Outcomes.

Study	Population (n)	Intervention Category	Intervention	Duration	Psychological and Stress-Related Outcomes	Microbiome Outcomes
Li et al. (2024) [63]	Healthy adults under mild stress (*n* = 120)	Psychobiotic	*Lactobacillus paracasei* 207-27	28 days	Increased sleep duration; reduced cortisol and HPA-axis activity	Increased Bacteroidota, *Bacteroides*, and *Megamonas*; reduced Firmicutes/Bacteroidetes ratio and *Escherichia–Shigella*; increased SCFA levels
Li J. et al. (2024) [64]	Healthy adults with high mental stress (*n* = 120)	Psychobiotic	*Bifidobacterium breve* 207-1	28 days	Improved sleep quality; increased GABA and reduced HPA-axis hormone levels; no significant changes in mood scores	Altered gut microbiota composition; increased SCFAs in the low-dose group; slight reduction in α-diversity in the high-dose group
Li J. et al. (2024) [65]	Students under academic stress (*n* = 27)	Psychobiotic	Heat-killed *Enterococcus faecalis* EC-12	7 days	No significant changes in mental symptoms or salivary cortisol	Increased *Enterococcus faecalis* abundance and fecal tryptamine; no significant changes in overall microbiota composition or diversity
Kim et al. (2021) [66]	Community-dwelling healthy older adults ≥65 years (*n* = 63)	Psychobiotic	*Bifidobacterium bifidum* BGN4 + *Bifidobacterium longum* BORI	12 weeks	Improved mental flexibility and reduced stress scores; increased serum BDNF	Reduced *Eubacterium*, *Allisonella*, Clostridiales, and Prevotellaceae; no significant changes in overall microbial diversity
Lee et al. (2021) [67]	Healthy adults with subclinical depression, anxiety, and insomnia (*n* = 156)	Psychobiotic	NVP-1704 (*Lactobacillus reuteri* NK33 + *Bifidobacterium adolescentis* NK98)	8 weeks	Reduced depressive and anxiety symptoms; improved sleep quality; reduced IL-6, with no significant changes in cortisol or ACTH	Increased Bifidobacteriaceae and Lactobacillaceae; decreased Enterobacteriaceae
Tie et al. (2025) [68]	Adults with anxiety and/or depressive symptoms (*n* = 79)	Psychobiotic	*Weizmannia coagulans* BC99	8 weeks	Reduced anxiety and depressive symptom scores, but not significantly versus placebo; increased GABA and NO	Increased *Faecalibacterium*, *Megamonas*, *Dialister*, and *Agathobacter*; increased SCFA production; no significant change in overall microbial diversity
Nishida et al. (2019) [69]	Medical students under examination stress (*n* = 60)	Psychobiotic	Heat-inactivated *Lactobacillus gasseri* CP2305	24 weeks	Reduced anxiety and sleep disturbance; improved objective sleep parameters; reduced salivary chromogranin A	Attenuated stress-associated changes in gut microbiota composition and diversity
Kato-Kataoka et al. (2016) [70]	Healthy medical students under examination stress (*n* = 47)	Psychobiotic	Fermented milk containing *Lactobacillus casei* Shirota	8 weeks	Attenuated increase in perceived stress; prevented significant increase in salivary cortisol; reduced stress-related abdominal symptoms	Preserved gut microbial diversity; increased species richness and reduced Bacteroidaceae compared with placebo
Horvath et al. (2026) [71]	Adults with impaired sleep quality (*n* = 94)	Psychobiotic	OMNi-BiOTiC^®^ STRESS Repair multispecies probiotic	28 days	Improved sleep quality, sleep efficiency, and sleep latency; no significant effect on perceived stress or quality of life	Partial recovery of administered probiotic bacteria; slight shift in β-diversity
Vázquez-Castellanos et al. (2025) [72]	Healthy students under academic stress (*n* = 79)	Psychobiotic	Fermented milk containing *Lacticaseibacillus rhamnosus* CNCM I-3690	4 weeks	Reduced perceived stress; lower anxiety showed a non-significant trend in the analyzed cohort	Attenuated stress-associated changes in α- and β-diversity; maintained *Ruminococcus bicirculans* abundance; higher *Faecalibacterium prausnitzii* abundance was associated with lower anxiety
Bloemendaal et al. (2021) [73]	Healthy women (*n* = 56)	Psychobiotic	Ecologic^®^ BARRIER multispecies probiotic	28 days	Protected against stress-induced deterioration in working memory performance	No significant intervention effect on overall β-diversity or taxa after multiple-comparison correction; increased Ruminococcaceae_UCG-003 abundance was associated with greater protection of working memory under stress
Ahmad et al. (2025) [74]	Adults with sleep disturbances and depressive or anxiety symptoms (*n* = 99)	Psychobiotic	Circadian-supporting probiotic formulation	12 weeks	Reduced depressive and anxiety symptoms; improved sleep efficiency and REM sleep; reduced IL-6 and TNF-α	Increased microbial diversity, *Lactobacillus* and *Bifidobacterium abundance*, and SCFA-producing bacteria; increased butyrate and propionate
Wu et al. (2026) [75]	Infertile women with leuprorelin-induced perimenopausal syndrome (*n* = 100)	Psychobiotic	*Limosilactobacillus reuteri* NCU-37	4 weeks	Reduced anxiety, depressive symptoms, insomnia, and perimenopausal symptom severity; increased E2, AMH, FSH, and LH levels	Altered gut microbiota composition and diversity; increased *Blautia* and reduced *Bacteroides* abundance
Eastwood et al. (2025) [76]	Healthy older adults (*n* = 30)	Psychobiotic	Ecologic^®^ BARRIER multispecies probiotic	8 weeks	Improved cognitive reactivity to sad mood, including reduced hopelessness, rumination, and aggression; no significant effects on perceived stress, depression, or anxiety	No significant changes in α-diversity, β-diversity, richness, or overall taxonomic composition; increased relative abundance of *Lactococcus*
Márquez-Morales et al. (2021) [77]	Medical students under academic stress (*n* = 45)	Psychobiotic	Aguamiel-based beverage fermented with *Lactobacillus plantarum*, *L. paracasei*, and *L. brevis*	8 weeks	Reduced academic stress, including environmental demands and physical and psychological stress-related symptoms	Increased Firmicutes and Bacteroidetes; no significant change in Gammaproteobacteria
Wang et al. (2024) [78]	College students with diarrhea (*n* = 100)	Psychobiotic	*Bifidobacterium breve* BB05	2 weeks	Reduced anxiety and depressive symptoms; increased fecal 5-HT and reduced acetylcholine, epinephrine, and norepinephrine	Increased gut microbial diversity; partially restored microbiota composition, including increased *Bifidobacterium* and *Roseburia*
Liu et al. (2025) [79]	Patients with mild-to-moderate ulcerative colitis and anxiety and/or depression (*n* = 60)	Psychobiotic	Probiotic LAB + conventional mesalazine treatment	8 weeks	Significantly greater anxiety improvement; greater improvement in depression did not reach statistical significance	Increased relative abundance of Firmicutes; no significant microbiota changes in the control group
Wang et al. (2024) [80]	Patients with functional dyspepsia (*n* = 95)	Psychobiotic	Chaihu-Shugan-San (CSS)	4 weeks	Reduced anxiety and depressive symptoms; improved quality of life	Altered gut microbiota composition and relative abundance of specific bacterial taxa
Berding et al. (2023) [81]	Healthy adults with poor dietary habits (*n* = 45)	Dietary	Psychobiotic diet rich in prebiotic and fermented foods	4 weeks	Reduced perceived stress within the intervention group, but not significantly versus control; biological stress markers were not significantly affected	Subtle changes in microbial composition and function; altered fecal lipid and urinary tryptophan metabolite profiles; greater microbial stability was associated with greater reductions in perceived stress
Herselman et al. (2022) [82]	Healthy undergraduate students exposed to academic stress (*n* = 80)	Dietary	Walnuts (56 g/day)	16 weeks	Improved mental health and mood; protected against some stress-related increases in cortisol and α-amylase, with sex-dependent effects	Attenuated stress-associated reduction in gut microbial diversity in females; microbiome effects in males could not be established
Chen et al. (2023) [83]	Patients with type 2 diabetes mellitus (*n* = 17)	Dietary	High-fiber diet containing whole grains, prebiotics, and traditional Chinese medicinal foods	8 weeks	Reduced anxiety and depressive symptoms; reduced serum IL-1β, IL-6, MCP-1, and TNF-α	Increased microbial richness and diversity; increased *Lactobacillus*, *Akkermansia*, *Bifidobacterium*, *Bacteroides*, *Ruminococcus*, and *Blautia*; decreased Firmicutes/Bacteroidota ratio, Proteobacteria, and several opportunistic pathogens
Crichton et al. (2023) [84]	Healthy young adults aged 18–30 years (*n* = 51)	Dietary	Standardized ginger (*Zingiber officinale*) root powder (1.2 g/day)	14 days	No significant effects on depression, anxiety, stress, fatigue, or quality of life	Increased Actinobacteria and greater abundance of *Parabacteroides*, *Bacillus*, and Ruminococcaceae incertae sedis; reduced *Blautia*; no significant changes in α- or β-diversity
Gillies et al. (2024) [85]	Healthy women aged 18–45 years (*n* = 40)	Dietary	Flavonoid-rich blackcurrant beverage (151 mg anthocyanins; 308 mg total polyphenols)	4 weeks	Reduced tension/anxiety and anger/hostility; improved working memory; no significant effect on stress reactivity, sleep, BDNF, tryptophan, kynurenine, or tryptophan/kynurenine ratio	No significant effects on α-diversity, β-diversity, microbial composition, or functional potential; higher baseline *Bifidobacterium*, *B. longum*, and *B. bifidum* abundance was associated with greater working-memory improvement
Jackson et al. (2023) [86]	Healthy working adults with mild-to-moderate anxiety and depression (*n* = 92)	Dietary	Oligofructose (8 g/day), 2′-fucosyllactose (2 g/day), or their combination	5 weeks	Oligofructose reduced anxiety and improved positive and negative mood; combined oligofructose/2′-fucosyllactose improved positive mood; no significant effects on cortisol awakening response	Oligofructose and the combined intervention increased *Bifidobacterium*, *Bacteroides*, *Roseburia*, and *Faecalibacterium prausnitzii*; 2′-fucosyllactose produced distinct microbiome responses
Marx et al. (2026) [87]	Healthy women aged 18–55 years (*n* = 40)	Dietary	Probiotic fermented yogurt (130 g/day)	8 weeks	No significant effects on mental health, memory, or blood markers of oxidative stress and inflammation; preliminary changes in hippocampal glutathione and functional connectivity	Significant change in gut microbiome β-diversity; no significant differences in other microbiome measures
Yang et al. (2026) [88]	Perimenopausal women with neuropsychiatric symptoms (*n* = 64)	Dietary	Flaxseed lignan extract (50 mg SDG/day) + health education	1 month	Reduced depressive and anxiety symptoms; increased serum estradiol	Increased fecal enterolactone, enterodiol, and β-glucuronidase; enrichment of specific gut bacteria, particularly *Bacteroides ovatus*
Selma-Royo et al. (2025) [89]	Pregnant women at high risk of delivering a small-for-gestational-age newborn (*n* = 351)	Other lifestyle-oriented	Mediterranean diet supplemented with extra-virgin olive oil and walnuts or MBSR	Pregnancy intervention (from 19–23 to 34–36 gestational weeks)	MBSR targeted maternal stress reduction; psychological effects were established in the parent IMPACT BCN trial.	Mediterranean diet altered overall gut microbiota structure, increasing Firmicutes, Lachnospiraceae/Ruminococcaceae and other SCFA producers and reducing *Campylobacter*; MBSR showed more limited microbiome effects
Bellach et al. (2024) [90]	Women with overweight/obesity (*n* = 41)	Other lifestyle-oriented	Caloric restriction (very-low-calorie diet or F.X. Mayr diet), with or without CPI	2 weeks	Improved perceived stress and other psychometric outcomes following caloric restriction; CPI provided little additional benefit	Reduced α-diversity and significant shifts in β-diversity; decreased *Agathobacter*, *Fusicatenibacter*, and *Subdoligranulum*; *Oscillibacter* enrichment occurred only with the F.X. Mayr diet and was associated with improved psychometric outcomes; CPI had negligible microbiome effects
Tunali et al. (2024) [91]	Patients with irritable bowel syndrome (*n* = 121)	Other lifestyle-oriented	Microbiome-based AI-assisted PD or LFD	6 weeks	Both diets significantly reduced anxiety and improved quality of life; no significant improvement in depression	PD induced significant shifts in microbial diversity and taxonomic composition compared with LFD

**Abbreviations:** ACTH, adrenocorticotropic hormone; AI, artificial intelligence; AMH, anti-Müllerian hormone; BDNF, brain-derived neurotrophic factor; CPI, clinical psychological intervention; E2, estradiol; FSH, follicle-stimulating hormone; GABA, γ-aminobutyric acid; HPA, hypothalamic–pituitary–adrenal; IL, interleukin; LAB, lactic acid bacteria; LFD, low-FODMAP diet; LH, luteinizing hormone; MBSR, Mindfulness-Based Stress Reduction; MCP-1, monocyte chemoattractant protein-1; NO, nitric oxide; PD, personalized diet; REM, rapid eye movement; SCFA(s), short-chain fatty acid(s); SDG, secoisolariciresinol diglucoside; TNF-α, tumor necrosis factor-alpha.

**Table 3:** Summary of the Main Findings Across Intervention Categories.

Intervention Category	No. of RCTs	Main Psychological Outcomes	Main Gut Microbiome Outcomes	Key Biological Findings	Overall Evidence
Psychobiotic interventions	18	Frequent but non-uniform improvements in perceived stress, anxiety, sleep, mood, and selected cognitive outcomes	Preservation of gut microbial diversity during psychological stress; increased abundance of *Bifidobacterium*, *Faecalibacterium prausnitzii*, and members of the phylum Firmicutes; favorable alterations in overall gut microbial composition	Reduced cortisol concentrations; increased GABA levels; evidence of gut–brain axis modulation	Generally favorable but heterogeneous evidence, with several null or outcome-specific findings and substantial strain-specific variability.
Dietary interventions	8	Reduced perceived stress; improved mood, emotional well-being, and quality of life; reduced anxiety and depressive symptoms in selected populations	Increased microbial diversity; preservation of gut microbial richness; increased abundance of *Lactobacillus*, *Bacteroides ovatus*, and other beneficial taxa; favorable alterations in gut microbial composition	Altered tryptophan metabolism; modulation of gut–brain axis-related biomarkers; microbiome-associated metabolic changes	Mixed but potentially beneficial evidence; psychological and microbiome effects varied substantially according to intervention type, population, and outcome assessed.
Other lifestyle-oriented/personalized nutrition interventions	3	Reduced perceived stress and anxiety and improved psychological or quality-of-life outcomes in selected populations	Increased abundance of Lachnospiraceae and Ruminococcaceae; improved gut microbial diversity; favorable alterations in β-diversity	Modulation of microbial diversity, SCFA-associated taxa, and microbiome-related pathways across heterogeneous lifestyle and personalized nutrition approaches	Promising but heterogeneous evidence; intervention designs differed substantially and do not support conclusions regarding the superiority of combined approaches.

**Table 4:** Risk of Bias Assessment of the Included Randomized Controlled Trials Using the Cochrane Risk of Bias 2 (RoB 2) Tool.

Study	D1	D2	D3	D4	D5	Overall
Li et al. (2024) [63]	Low	Low	Low	Low	Low	Low
Li J. et al. (2024) [64]	Low	Low	Low	Low	Low	Low
Li J. et al. (2024) [65]	Low	Low	Low	Low	Low	Low
Kim et al. (2021) [66]	Low	Low	Low	Low	Low	Low
Lee et al. (2021) [67]	Low	SC	SC	Low	Low	SC
Tie et al. (2025) [68]	Low	Low	Low	Low	Low	Low
Nishida et al. (2019) [69]	Low	Low	Low	Low	Low	Low
Kato-Kataoka et al. (2016) [70]	Low	Low	Low	Low	Low	Low
Horvath et al. (2026) [71]	Low	SC	SC	Low	Low	SC
Vázquez-Castellanos et al. (2025) [72]	Low	Low	Low	Low	Low	Low
Bloemendaal et al. (2021) [73]	Low	Low	Low	Low	Low	Low
Ahmad et al. (2025) [74]	Low	Low	Low	Low	Low	Low
Wu et al. (2026) [75]	Low	Low	Low	Low	Low	Low
Eastwood et al. (2025) [76]	Low	Low	Low	Low	Low	Low
Márquez-Morales et al. (2021) [77]	High	SC	Low	High	SC	High
Wang et al. (2024) [78]	Low	Low	Low	Low	Low	Low
Liu et al. (2025) [79]	Low	SC	Low	High	Low	High
Wang et al. (2024) [80]	Low	Low	Low	Low	SC	SC
Berding et al. (2023) [81]	Low	Low	Low	Low	Low	Low
Herselman et al. (2022) [82]	SC	High	Low	High	Low	High
Chen et al. (2023) [83]	Low	Low	Low	SC	SC	SC
Crichton et al. (2023) [84]	Low	Low	Low	Low	Low	Low
Gillies et al. (2024) [85]	Low	Low	Low	Low	Low	Low
Jackson et al. (2023) [86]	Low	Low	Low	Low	Low	Low
Marx et al. (2026) [87]	Low	Low	Low	Low	Low	Low
Yang et al. (2026) [88]	Low	Low	Low	Low	Low	Low
Selma-Royo et al. (2025) [89]	Low	Low	Low	Low	Low	Low
Bellach et al. (2024) [90]	Low	High	Low	SC	SC	High
Tunali et al. (2024) [91]	Low	Low	High	High	Low	High

**Abbreviations:** D1, Bias arising from the randomization process; D2, Bias due to deviations from intended interventions; D3, Bias due to missing outcome data; D4, Bias in measurement of the outcome; D5, Bias in selection of the reported result. Low = Low risk of bias; SC = Some concerns regarding risk of bias; High = High risk of bias.

## Data Availability

No new data were created or analyzed in this study. All data supporting the findings of this systematic review are available within the published articles included in the review and the information presented in this manuscript.

## Supplementary Materials

The following supporting information can be downloaded at: https://www.mdpi.com/article/10.3390/nu18172909/s1, Table S1. PRISMA 2020 checklist. Table S2. Complete database-specific search strategies.

## Abbreviations

The following abbreviations are used in this manuscript:

HPA	Hypothalamic–pituitary–adrenal
GABA	γ-aminobutyric acid
SCFAs	Short-chain fatty acids
PRISMA	Preferred Reporting Items for Systematic Reviews and Meta-Analyses
RCTs	Randomized controlled trials
